# Beyond Food Access: The Impact of Parent-, Home-, and Neighborhood-Level Factors on Children’s Diets

**DOI:** 10.3390/ijerph14060662

**Published:** 2017-06-20

**Authors:** Lauren Futrell Dunaway, Thomas Carton, Ping Ma, Adrienne R. Mundorf, Kelsey Keel, Katherine P. Theall

**Affiliations:** 1 Department of Global Community Health and Behavioral Sciences, Tulane School of Public Health and Tropical Medicine, New Orleans, LA 70112, USA; lfutrell@tulane.edu (L.F.D.); kkeel@tulane.edu (K.K.); 2 Mary Amelia Douglas Whited Community Women’s Health Education Center and Prevention Research Center (PRC), Tulane School of Public Health and Tropical Medicine, New Orleans, LA 70112, USA; 3 Louisiana Public Health Institute, New Orleans, LA 70112, USA; tcarton@lphi.org; 4 Children’s Health^SM^, Children’s Medical Center, Dallas, TX 75235, USA; maping76@gmail.com; 5 Sisters of Charity Foundation of Cleveland, Cleveland, OH 44115, USA; amundorf@socfcleveland.org

**Keywords:** neighborhood, children, diet, family

## Abstract

Despite the growth in empirical research on neighborhood environmental characteristics and their influence on children’s diets, physical activity, and obesity, much remains to be learned, as few have examined the relationship between neighborhood food availability on dietary behavior in children, specifically. This analysis utilized data from a community-based, cross-sectional sample of children (*n* = 199) that was collected in New Orleans, Louisiana, in 2010. This dataset was linked to food environment data to assess the impact of neighborhood food access as well as household and parent factors on children’s diets. We observed a negligible impact of the neighborhood food environment on children’s diets, except with respect to fast food, with children who had access to fast food within 500 m around their home significantly less likely (OR = 0.35, 95% CI: 0.1, 0.8) to consume vegetables. Key parental and household factors did play a role in diet, including receipt of public assistance and cooking meals at home. Children receiving public assistance were 2.5 times (95% CI: 1.1, 5.4) more likely to consume fruit more than twice per day compared with children not receiving public assistance. Children whose family cooked dinner at home more than 5 times per week had significantly more consumption of fruit (64% vs. 58%) and vegetables (55% vs. 39%), but less soda (27% vs. 43%). Findings highlight the need for future research that focuses on the dynamic and complex relationships between built and social factors in the communities and homes of children that impact their diet in order to develop multilevel prevention approaches that address childhood obesity.

## 1. Introduction

Childhood obesity prevalence rates in the United States (U.S.) have remained stable between 2003 and 2004 and between 2009 and 2010, but the number of children who become obese at younger ages is increasing, and as many as 80% of obese children and adolescents will become obese adults [1]. Since the 1970s in the United States, this percentage has more than tripled [2], and 20% of school-aged children are obese [3]. While research has shown that it is difficult to predict trends for childhood obesity [3], one study estimates that, for adults, if obesity prevalence increases at the rate that it has between 1990 and 2008, there could be a 33% increase for obesity and a 130% increase for severe obesity by 2030 in the U.S. [4]. Obese and overweight children face numerous health problems that can extend into adulthood including sleep apnea, orthopedic complications, negative psychosocial interactions, type 2 diabetes, and hypertension; most of which were not previously common in children [5]. These are in addition to chronic diseases exacerbated by or seen as comorbidities with obesity such as heart disease, cancer, chronic kidney disease, asthma, and arthritis to name a few [6]. Experts agree that multilevel, comprehensive obesity prevention strategies are needed to understand and address this serious public health concern [7].

Despite the growth in empirical research on neighborhood environmental characteristics and their influence on children’s diets, physical activity, and obesity [8,9,10], much remains to be learned, as most work in childhood obesity has focused on the micro-environment (e.g., home and school influences) [11,12,13,14] and few have examined the relationship between neighborhood food availability on dietary behavior in children specifically [12,15,16,17,18]. Neighborhood contextual factors are likely to have an impact on an individual’s weight through direct or indirect influences on food intake and/or physical activity [13,14]. 

Neighborhood factors that influence child dietary behaviors need to be better understood to develop effective nutrition interventions. The physical availability of unhealthy food choices (e.g., density of fast food restaurants, and convenience stores) may be an important contributor to poor dietary habits and the risk of overweight [19,20,21]. Consumption of high-fat fast food may contribute to higher energy and fat intake, and lower intake of healthful nutrients [8,22], which could increase the risk of obesity because of massive portion sizes, high energy density, and palatability. Furthermore, the neighborhood nutrition environment may help to explain some of the racial, ethnic, and socioeconomic disparities in dietary patterns [14,23]. Fast food restaurants have been shown to be more prevalent in neighborhoods with a greater population of non-white individuals, where supermarkets are less prevalent [24,25,26]. Similarly, low-income neighborhoods have reportedly lower availability to healthy foods [27] and decreased energy adjusted intake of fruits, vegetables, and fish, with an increased intake of meat [28] than high-income neighborhoods. 

While neighborhood level factors remain important, analyses of a U.S. national survey of children found that differential exposure to food outlets does not independently explain weight gain over time in elementary school-aged children and that variation in food outlet availability does not explain socioeconomic and racial/ethnic differences [29]. The placements of new supermarkets in underserved communities have shown to moderately improve residents’ perceptions of food accessibility, but do not lead to changes in body mass index, reported increase of fruit and vegetable intake [30,31], or the availability of healthful foods at home or in children’s dietary intake [16]. It may be crucial to consider other factors, beyond neighborhood food access, when trying to understand obesity risk among children.

The New Orleans, Louisiana (U.S.), area is rapidly changing and developing, making it an important city in which to examine the structure and significance of neighborhoods and health promotion activities. Children and families in New Orleans are undergoing alterations in their community environments and have been, and continue to be, exposed to heightened socioemotional stress, referred to as solastalgia, as a result of Hurricane Katrina [32,33]. Geographic food access has been shown to differ for black individuals in New Orleans, with fewer supermarkets (which have been associated with lower BMI (body mass index) and healthier food consumption) [34] and less availability of fresh fruits and vegetables in predominantly black or African-American neighborhoods [35]. Recent analyses indicate that this disparity increased after Katrina in 2007, and the disparity in access returned to pre-Katrina levels in 2009, but there remained a disproportionately unhealthy food environment for black individuals in New Orleans [35,36,37,38,39].

A multilevel, community-based study afforded the opportunity to examine the impact of local food access on diet among a cross-sectional sample of children, while taking into consideration multiple family, and individual level factors.

## 2. Materials and Methods

As part of a study to examine neighborhood influences on children’s health disparities, 199 children ages 4–14 years and their families were recruited from January–May 2010 through five local schools in an inner-city community in New Orleans, Louisiana. Schools who participated in the study served children from pre-kindergarten (Pre-K 4) to eighth grade. Data were collected at the level of the child, family/household, and neighborhood using a caregiver survey including the residential address of the child, which was geocoded. While children were recruited from schools within the same community, 87 census tracts were represented residentially.

Families were recruited through the schools via active consent with letters and a quantitative survey for caregivers sent home with the child and returned through the school. The caregiver survey assessed numerous factors, including obesogenic factors in the household, racial discrimination and child-specific data such as behavioral and emotional problems and other confounders (e.g., prenatal exposure and events). Families also reported if they were the recipients of food-related public assistance programs such as the Supplemental Nutrition Assistance Program (SNAP), formerly the Food Stamp Program, and Women, Infants, and Children (WIC). Children in this study came from unique households, with no sibling groups represented in the sample. Caregivers were also asked to provide the residential address of the child, which were geocoded to plot the child within their census tract. The project staff and school nurse were available (via phone or in person) to assist the caregivers in completing the quantitative survey. Additionally, as part of a larger study anthropometric measures, including height, weight, blood pressure, and waist circumference were collected on each child, at the school, by trained staff using standardized techniques. However, these were collected by partner group and anthropometric items were not available for this study.

A multilevel data system of children nested within their household (HH) and neighborhood was created. Sources for neighborhood data included the U.S. Census 2000, geographic data from ArcGIS, U.S. Census shapefiles, and food environment data from the Tulane Prevention Research Center (PRC). Definitions of neighborhood were based on the Census 2000 TIGER shapefile. Food store data collected included type of store (supermarket or small) based on the percentage of the stores total gross grocery sales [36], and 0.5-, 1.0-, and 2.0-mile distances from the child’s house to the store were calculated. 

The primary outcomes of interest were consumption of food (fruit, vegetables, and sweets) and beverages (fruit juice, milk, and soda). Key dietary components were included based on common components in national surveys in the U.S. and balanced with respondent burden given the number of questions asked. Fruit juice, soda, and sweets were considered obesogenic factors in this study [40,41,42,43]. Parents reported on consumption of servings per day for each food and beverage group, and all consumption measures were coded to be dichotomous. Fruit, vegetables, juice, and milk were coded “1” if children consumed two or more servings per day, on average, and “0” if children consumed less than two servings per day. Sweets and soda were coded “1” if children consumed at least one serving per day, on average, and “0” if children consumed less than one serving per day. 

The primary exposure of interest was a food store within a given perimeter of the child’s household (500 m for small food stores and 1000 m for supermarkets). Descriptive univariate, bivariate, and multivariate analyses were conducted. Based on previous food consumption literature, variables regarding age, sex, maternal marital status and education, number of children in the household, reception of public assistance, and dinners consumed at home were controlled for. Logistic regression models for individual food and beverage consumption outcomes were constructed using STATA 10.0 (STATA, College Station, TX, USA), with hierarchical models that accounted for census tract or neighborhood-level clustering of children living within the same neighborhood. 

## 3. Results

Descriptive analysis on varied food and beverage consumption by individual, family, and neighborhood’s characteristics and bivariate analysis on association between independent variables and food and beverages consumption are presented in Table 1, except for race. The sample was 97% African-American or black with the remaining 3% being white or of other races. Among the 3% of children in the sample of races other than black, there were less than 5 children in each additional race category, so race is not presented and was not included in further analyses. Significant differences in food and beverage consumption were found across individual demographic variables, including child’s age, sex, and mother’s educational degree. Approximately 65% of sampled children aged 6 years old or younger consumed fruit more than twice per day compared with 60% of children aged 7–9 years old and 46% of children aged 10 or older. For juice consumption, over 80% of children 6 years old or younger had juice more than twice per day, while 70% of children aged 7–9 years old and 56% of children aged 10 or older had juice more than twice per day. The significant differences between fruit consumption and juice across children’s age groups were found in bivariate analysis. There were no significant differences in the consumption of vegetables, sweets, milk, or soda by age. 

Differences in the consumption of fruit and sweets by sex were demonstrated in the bivariate analysis, with 62% of male children versus 50% of female children having access to fruit more than twice per day and 58% of male versus 70% of female children consuming sweets more than twice per day. No significant sex differences were shown in the consumption of vegetables, milk, and soda. 

There was no significant difference in the frequency of any food or beverage consumption based on the mother’s marital status. However, the mother’s education level was found to significantly influence children’s consumption on sweets and juice, milk (although marginally, *p* < 0.10), and soda. Approximately 71% of children whose mothers had a high school education consumed sweets more than twice per day, compared with 67% of children whose mothers with less than a high school education, and 47% whose mothers had more than a high school education. Greater proportions of children with mothers who had lower education consumed juice, milk, and soda more than twice per day compared to those whose mothers had higher education. 

Despite no significant finding on the relationship between the consumption of food or beverages and the number of children in family, significant associations were found between two other family environment-related variables and food or beverage consumption. Children who received public assistance had a significantly higher likelihood of consuming fruit, vegetables, and juice more than twice per day (*p* < 0.05) than children who did not receive any public assistance. Consistent with previous findings, children whose family cooked dinner at home more than 5 times per week had significantly more consumption of fruit (64% vs. 58%) and vegetables (55% vs. 39%), but less soda (27% vs. 43%) compared to children whose family cooked dinner at home less than 4 times per week. 

With respect to the three neighborhood variables, access to a small store within 500 m and supermarket within 1000 m around residency were not significantly associated with children’s food and beverage consumption. However, children who had access to fast food within 500 m from their home were significantly less likely to consume fruit (43% vs. 59%) and vegetables (24% vs. 50%) than children with less fast food access. 

Table 2 shows the multivariate analysis of the association between the neighborhood food environment and selected food and beverage consumption. Results reveal that children who had access to fast food within 500 m around their home were nearly three times less likely (OR = 0.35, 95% CI: 0.1, 0.8) to consume vegetables when controlling for individual demographic characteristics and family relevant variables. Cooking dinner at home and family receiving public assistance remained significantly associated with children’s vegetables consumption after controlling for other potential confounders. Compared with the children from families who cook dinner at home less than 4 times per week, the children from families who cook dinner more than 5 times per week were nearly two times more likely to consume vegetables (OR = 1.9, 95% CI: 1.1, 3.5). Children receiving any public assistance were also more than twice as likely to have access to vegetables compared to children not receiving any public assistance after controlling for other potential confounders (OR = 2.3, 95% CI: 1.2, 4.5). No significant relationships were observed for fruit and sweets consumption. 

In addition, children aged 10–14 years old and children with high school educated mothers have lower likelihoods of drinking juice more than twice per day (OR = 0.2, 95% CI: 0.1, 0.5; OR = 0.4, 95% CI: 0.2, 0.8) than children aged 4–6 years old and children whose mothers have less than a high school education, respectively. Children receiving public assistance were 2.5 times (95% CI: 1.1, 5.4) more likely to consume fruit more than twice per day compared with children not receiving public assistance. Children whose mother had an education level greater than high school were less likely to drink milk more than twice per day compared to children with an education level lower than high school in the adjusted model (OR = 0.3, 95% CI: 0.1, 0.7). Regarding the consumption of soda, children with high school educated mothers, and those from a family who cooked dinner more than five times per week were less likely to drink soda more than once per day, compared with children with less educated mothers (OR = 0.4, 95% CI: 0.2, 0.8) and children from families who cook less than 4 times per week (OR = 0.5, 95% CI: 0.2, 0.9).

## 4. Discussion

The majority of research examining the relationship between multi-level factors related to childhood obesity have traditionally focused on the home and school. This study aimed to contribute to the limited body of literature focusing on this relationship at the neighborhood level. While significant differences in food and beverage consumption were found for certain individual and maternal demographic variables, at the neighborhood level, the presence of a fast food restaurant near the child’s home was the only neighborhood level exposure significantly associated with decreased vegetable consumption. Existing research that has examined the association between fast food availability and childhood obesity is mixed with significant associations found among adolescents [44], but not among preschool aged children [45]. The presence of a food store within a radius of the child’s household was not significantly correlated with consumption, and this is consistent with existing research that has documented an inverse or null association between the presence of supermarket and dietary behavior in children [12,15,16,17,18].

Although the relationship between the presence of a food store and consumption was not significant in these multivariable analyses, there seem to be two key factors affecting this relationship—maternal education and meals consumed at home. While the influence of the home environment on childhood obesity has been established, there are many factors that may be impacting this relationship [46]. An increase in the number of meals consumed away from home has been shown to be significantly associated with an increased energy intake of children [47]. Additionally, when children and adolescents consume meals away from home, they are more likely to consume sugar-sweetened beverages [48]. While experts agree that maternal education is an important factor to consider in regard to childhood obesity, some existing studies have not found the two to be significantly associated [49,50], while others suggest that the relationship between the two may be mediated by income [46]. Receipt of public assistance was also associated with an increase in vegetable intake, which contributes to mixed findings on the impact of both the Supplemental Nutrition Assistance Program (SNAP) and Women, Infants, and Children (WIC). Some studies have shown that participation in WIC resulted in increased nutrient intake for preschool children [51], but other studies have shown no difference in preschool children’s diet quality or weight status based on SNAP or WIC participation [52,53].

Excessive juice consumption was also a point of interest in this study. While younger children were more likely to consume juice daily than older children, it has been suggested that, as children age, they may be replacing beverages such as juice and milk with soft drinks [54], and although we did not see this pattern in this sample, it can be argued that, while juice and soft drinks are both obesogenic factors, soft drinks have even less nutritional value. Furthermore, children with mothers with less than a high school education were more likely to consume both juice and soda, and this association between maternal education and child feeding behaviors has been documented in the literature [55,56].

Further research is needed that captures the multiple levels that may influence dietary behavior in children and, in turn, childhood obesity, but research that examines the pathways through which these factors act is also needed. This was not possible in the current cross-sectional study. Identification of family level factors that mediate the relationships between neighborhood food access or level of food availability will also be crucial for identifying programs and policies to address the childhood obesity epidemic [56], particularly those that address and involve parents [57]. Mediators such as parent stress [58,59], parent employment [59,60], transportation [13,61], and cultural preferences and barriers are important to consider [62,63].

Limitations for this study include the cross-sectional nature of the design, the small sample size, and the lack of comprehensive dietary measures. Dietary behavior was based on parent report and may be subject to recall and misclassification bias, and a dichotomous categorization of the child’s neighborhood food environment may be too simplistic to characterize the effects of food availability on consumption [37]. Despite these limitations, this study sheds light on the complex relationship between neighborhood food availability and dietary behavior among children and within households.

## 5. Conclusions

Study findings reveal that parent, home, and neighborhood level factors may have an impact on children’s diets—particularly parent education, household receipt of public assistance, cooking meals at home, and neighborhood fast food access. The complex relationship between the built and social factors in both the communities and homes of children and their impact on obesity must be taken into account in developing multilevel prevention approaches. A more detailed, multi-level examination of the nutrition environment may help explain some of the racial, ethnic, and socioeconomic disparities in dietary patterns [64]. These results are important for continued work in New Orleans, where, although environmental food access has improved, a disproportionate number of underserved neighborhoods with unhealthy food environments still exist [39].

## Figures and Tables

**Table 1:** Food consumption by individual, family, and neighborhood characteristics (*N* = 199).

Independent Variables	*n*	Food %			Beverages %		
		Fruit (2+/Day)	Vegetables (2+/Day)	Sweets (1+/Day)	Juice (2+/Day)	Milk (2+/Day)	Soda (1+/Day)
I: Child: Age							
≤6	71	64.7% *	44.1%	64.7%	80.9% ***	52.9%	38.2%
7–9	40	59.6%	43.5%	68.1%	70.2%	52.2%	26.1%
≥10	58	46.4%	46.4%	61.9%	56.0%	39.8%	43.4%
I: Child: Sex							
Male	88	62.4% *	49.5%	58.1% *	66.7%	49.5%	35.5%
Female	111	50.0%	41.0%	69.8%	68.9%	45.2%	39.4%
HH: Mother: Marital status							
Not married cohabiting	159	58.5%	42.4%	62.9%	67.3%	49.4%	39.9%
Married cohabiting	40	45.0%	55.0%	70.0%	70.0%	38.5%	28.2%
HH: Mother: Education							
Less than high school	53	63.0%	48.2%	66.7% **	81.5% **	52.8% *	54.7% ***
High school (or GED ^†^)	52	53.0%	40.4%	71.0%	63.0%	51.0%	33.0%
More than high school	94	53.3%	51.1%	46.7%	62.2%	31.8%	27.3%
HH: Number of children							
1–2	127	54.1%	45.6%	64.2%	66.9%	45.9%	37.7%
3+	72	60.8%	43.1%	64.7%	70.6%	51.0%	37.3%
HH: Public assistance							
No	13	50.7% **	39.7% **	62.0%	62.7% **	47.9%	36.4%
Yes	186	68.4%	57.9%	70.2%	80.7%	45.6%	40.4%
HH: Cook dinner at home							
0–4 times/week	128	50.8% *	39.2% **	67.5%	66.7%	43.2%	43.2% **
5+ times/week	71	64.4%	54.8%	58.9%	69.9%	54.2%	27.8%
N: Small store within 500 m							
No	68	53.5%	47.9%	69.0%	66.2%	46.5%	36.6%
Yes	131	57.0%	43.3%	61.7%	68.8%	47.6%	38.1%
N: Supermarket within 1000 m							
No	145	54.9%	46.0%	66.1%	66.7%	48.1%	35.0%
Yes	54	59.5%	40.5%	56.8%	73.0%	43.2%	48.7%
N: Fast food within 500 m			49.7% ***				
No	146	58.6% *	24.3%	64.2%	68.5%	49.4%	36.9%
Yes	53	43.2%		64.9%	64.9%	37.8%	40.5%
Total		52.5%	44.4%	84.9%	65.0%	43.6%	65.0%

*** *p* < 0.01; ** *p* < 0.05; * *p* < 0.1; I: Individual, HH: Household, N: Neighborhood.; ^†^ GED: The General Education Development Test (GED) is a certificate in the U.S. equivalent to a high school diploma.

**Table 2:** Impact of the neighborhood food environment on food consumption: Multivariate model (adjusted odds ratios, 95% confidence interval) (*N* = 199).

Independent Variables	Food			Beverages		
	Fruits (2+/Day) (55.8%)	Vegetables (2+/Day) (45.0%)	Sweets (1+/Day) (64.3%)	Juice (2+/Day) (67.8%)	Milk (2+/Day) (47.2%)	Soda (1+/Day) (37.6%)
I: Child: Age						
4–6	-	-	-	-	-	-
7–9	0.6 (0.3, 1.4)	0.8 (0.3, 1.7)	1.0 (0.4, 2.2)	0.4 (0.2, 1.0)	0.8 (0.4, 1.7)	0.5 (0.2, 1.1)
10–14	0.4 (0.2, 0.9) **	1.2 (0.6, 2.3)	0.7 (0.4, 1.4)	0.2 (0.1, 0.5) ****	0.5 (0.3, 1.0)	1.2 (0.6, 2.3)
I: Child: Sex						
Male	-	-	-	-	-	-
Female	0.7 (0.4, 1.3)	0.8 (0.4, 1.5)	1.5 (0.8, 2.7)	1.5 (0.8, 2.8)	0.8 (0.4, 1.4)	1.1 (0.6, 2.1)
HH: Mother: Marital status						
Not married, cohabiting	-	-	-	-	-	-
Married, cohabiting	0.8 (0.4, 1.7)	2.0 (0.9, 4.1)	2.1 (1.0, 4.6)	2.3 (1.0, 5.2)	0.8 (0.4, 1.7)	0.8 (0.4, 1.8)
HH: Mother: Education						
Less than high school	-	-	-	-	-	-
High school (or GED)	0.8 (0.4, 1.5)	0.7 (0.4, 1.5)	1.1 (0.5, 2.3)	0.4 (0.2, 0.8) **	0.9 (0.5, 1.7)	0.4 (0.2, 0.8) ***
More than high school	0.6 (0.3, 1.5)	1.1 (0.5, 2.6)	0.4 (0.2, 1.0)	0.3 (0.1, 0.8) **	0.3 (0.1, 0.7) ***	0.4 (0.2, 1.0)
HH: Number of children						
1–2	-	-	-	-	-	-
3+	1.2 (0.6, 2.4)	0.8 (0.4, 1.5)	1.0 (0.5, 2.1)	1.2 (0.6, 2.6)	1.1 (0.6, 2.2)	1.0 (0.5, 2.0)
HH: Public assistance						
No	-	-	-	-	-	-
Yes	1.9 (1.0, 3.7)	2.3 (1.2, 4.5) **	1.7 (0.8, 3.4)	2.5 (1.1, 5.4) **	0.8 (0.4, 1.5)	1.4 (0.7, 2.7)
HH: Cook dinner at home						
0–4 times/week	-	-	-	-	-	-
5+ times/week	1.5 (0.8, 2.7)	1.9 (1.1, 3.5) **	0.7 (0.4, 1.3)	1.0 (0.5, 1.9)	1.7 (0.9, 3.1)	0.5 (0.2, 0.9) **
N: Small store						
No	†	†	†	†	†	†
Yes						
N: Supermarket						
No	†	†	†	†	†	†
Yes						
N: Fast food						
No	†	–	†	†	†	†
Yes		0.35 (0.1, 0.8) **				

† Food access variables were not significant in bivariate models and because key exposures of interest, therefore not included in multivariate analyses presented. **** *p* < 0.001; *** *p* < 0.01; ** *p* < 0.05; I: Individual, HH: Household, N: Neighborhood.
